# Prospective associations between childhood externalising and internalising problems and adolescent alcohol and drug use: The Bergen Child Study

**DOI:** 10.1177/1455072518789852

**Published:** 2018-08-08

**Authors:** Ove Heradstveit, Jens Christoffer Skogen, Tormod Bøe, Jørn Hetland, Mads Uffe Pedersen, Mari Hysing

**Affiliations:** Stavanger University Hospital, Norway; Uni Research Health, Bergen, Norway; Stavanger University Hospital, Norway; Norwegian Institute of Public Health, Bergen, Norway; Uni Research Health, Bergen, Norway; University of Bergen, Norway; Aarhus University, Denmark; Uni Research Health, Bergen, Norway

**Keywords:** adolescence, alcohol use, drug use, externalising problems, internalising problems, longitudinal

## Abstract

**Aims:**

The literature on associations between internalising problems and subsequent alcohol/drug use and problems shows mixed results, and it is important to consider different aspects of internalising problems along with co-occurring externalising problems.

**Methods:**

In a longitudinal study (*n* = 2438) followed up when the subjects were 7–9, 11–13, and 16–19 years of age, we investigated associations between parent/teacher-reported externalising and internalising problems (Strengths and Difficulties Questionnaire, SDQ) and adolescent self-reported alcohol and illicit drug use and problems. Socioeconomic status (SES), gender, and age were included as potential confounding variables. We also adjusted for the potential confounding effects from externalising problems on the association between internalising problems and alcohol/drug use, and vice versa.

**Results:**

Externalising problems were positively associated with all measures of alcohol/drug use and problems (adjusted odds ratios [*AOR*s] ranging from 1.24 to 1.40, all *p* < .05), while internalising problems were negatively associated with all measures of alcohol/drug use (*AOR*s ranging 0.83 to 0.88, all *p* < .05). Full-scale SDQ externalising problems were somewhat stronger and more robust predictors of adolescent alcohol/drug-related problems compared with SDQ externalising subscales, while only full-scale SDQ internalising problems were negatively associated with alcohol/drug-related problems. All estimates were similar across genders.

**Conclusions:**

Childhood externalising problems are positively associated while internalising problems are negatively associated with alcohol/drug use and problems in late adolescence.

Adolescence can be characterised by an escalation of alcohol and illicit drug use (Chassin, Sher, Hussong, & Curran, 2013), and the use of alcohol/drugs during adolescence may serve as a potent risk factor for both prolonged alcohol- and drug-related problems (Fergusson, Boden, & Horwood, 2008) and mental health problems (Marmorstein, 2009). However, the nature of the association between mental health problems and alcohol and drug use is complex, leading to suggestions of different etiological pathways and mechanisms (e.g., Chassin et al., 2013; Schulenberg & Maggs, 2002).

A range of previous publications demonstrate that childhood externalising problems – in which symptoms of deviancy, conduct problems, and hyperactivity/inattention are prominent – are important precursors to alcohol/drug use during adolescence (e.g., Fergusson, Horwood, & Ridder, 2007; Heron et al., 2013; Miettunen et al., 2014). However, previous literature on associations between childhood internalising problems – in which symptoms of depression, anxiety, peer problems, and social withdrawal are important – and adolescent alcohol/drug use is marked by a lack of consistent results. Some recent contributions have even pointed to a negative association between internalising problems and adolescent alcohol/drug use (Colder et al., 2013; Edwards et al., 2014; Scalco et al., 2014).

Previous findings highlight that a core feature in the externalising pathway towards substance use is behavioural disinhibition (Iacono, Malone, & McGue, 2008), whereas negative affect has been proposed as an important feature of internalising problems that may heighten the risk of hazardous alcohol/drug involvement (Hussong, Ennett, Cox, & Haroon, 2017; Hussong, Jones, Stein, Baucom, & Boeding, 2011). This is consistent with the broader self-medication hypothesis (e.g., Chassin et al., 2013; Khantzian, 1987), while internalising tendencies towards social withdrawal and fear of negative consequences are aspects suggested to decrease the risk of exposure to alcohol/drug use (Colder, Chassin, Lee, & Villalta, 2010; Hussong et al., 2011). Still, few empirical investigations have actually examined how specific internalising symptoms, such as peer/relationship problems and emotional problems, may affect alcohol/drug use, although these internalising factors may be differentially associated with adolescent alcohol/drug-related behaviours.

Importantly, comorbidity rates between internalising and externalising symptoms are high in childhood and adolescence (Chan, Dennis, & Funk, 2008), and it is likely that such high rates of comorbidity may obscure the unique associations between internalising symptoms and alcohol/drug-related problems (Hussong et al., 2011). It is therefore recommended that developmental models of internalising symptoms and alcohol/drug use should also consider externalising symptoms (Colder et al., 2018; Hussong et al., 2017). Although the majority of previous studies do not attempt to control for externalising symptoms when associations between internalising problems and alcohol/drug use are investigated (Hussong et al., 2011), a growing body of research has emerged complying with the recommendation to control for co-occurring externalising symptoms (for a review, see Hussong et al., 2017). For example, a study by Colder and colleges (2013) demonstrated that externalising problems in the absence of internalising problems yielded the strongest longitudinal association with both alcohol/drug use during early adolescence (12–16 years). For externalising problems in combination with internalising problems, a weak but statistically significant positive association with alcohol/drug use was found. Finally, internalising problems in the absence of externalising problems were associated with lower alcohol/drug use.

Our study uses the Strengths and Difficulties Questionnaire (SDQ) to investigate how childhood externalising and internalising problems precede alcohol/drug-related problems during late adolescence. We hypothesise that childhood externalising problems will have a robust positive association with adolescent alcohol/drug-related problems, and that childhood internalising problems are negatively associated with adolescent alcohol/drug-related problems, particularly after the adjustment of externalising problems. We also investigate how subtypes of externalising and internalising problems are differentially associated with alcohol/drug-related problems. We expected that both SDQ subscales of externalising problems (conduct problems and inattention/hyperactivity) are positively associated with alcohol/drug-related problems, and that the SDQ internalising subscale of peer/relationship problems is more strongly negatively associated with alcohol/drug-related problems compared with the SDQ internalising subscale of emotional problems, due to the conceptual proximity between peer/relationship problems and social withdrawal processes. Our study adds to the knowledge base with data from a large, longitudinal sample of Norwegian children.

## Methods

### Participants

The sample comprised participants from the Bergen Child Study (BCS; for more information about the BCS and related publications, see uni.no/en/bergen-child-study), and the data stem from the first, second, and fourth waves of this study. The BCS is a longitudinal total population study of children in all public and private schools in the city of Bergen, Norway. The fourth wave of the BCS is nested within the youth@hordaland survey (Sivertsen, Harvey, Pallesen, & Hysing, 2017) covering the whole Hordaland county, in which Bergen is the largest city, as its target population. The BCS has been utilised in a range of previously published studies, and has also recently been used in a longitudinal design (Sivertsen et al., 2017).

The first wave of the BCS, conducted in autumn 2002, comprised a target population of 9430 primary-school children aged 7–9 years from the city of Bergen, and informed consent to participate was received from 7007 parents (74%) prior to inclusion in the study. The second wave was conducted four years later during spring 2006, and 5683 children aged 11–13 years participated (60% of the original target population). Six years later in winter/spring 2012, when the adolescents were 16–19 years of age, the target population was expanded to include the whole county of Hordaland, and 10,253 (53%) of the 19,439 invited adolescents participated. The three waves used in our study are labelled T1, T2, and T3.

A total of 2438 adolescents had participated in T1, T2, and T3 with valid parent or teacher responses on the Strengths and Difficulties Questionnaire (SDQ) at both T1 and T2, and therefore comprised the final sample. The mean age of the total sample at T3 was 17.4 years (standard deviation 0.8), and 53.7% of the sample were girls.

The study was approved by the Regional Committee for Medical and Health Research Ethics in Western Norway.

### Explanatory variables: Childhood mental health problems

The variable of mental health problems was defined by scores on the Strengths and Difficulties Questionnaire (SDQ) (Goodman, 1997), which was completed separately by parents and teachers at T1 and T2. The SDQ is a screening questionnaire for children and adolescents aged 4–16 years, consisting of 25 items which describe positive and negative characteristics of children within five subscales: (1) emotional problems, (2) conduct problems, (3) hyperactivity/inattention problems, (4) peer/relationship problems, and (5) pro-social behaviour (not used in the current study). Each item is scored on a three-point scale – not true, somewhat true, and certainly true – with total subscale scores each ranging from 0 to 10.

The SDQ has been validated in several countries (Heiervang et al., 2007; Muris, Meesters, & van den Berg, 2003). A recent review found that the psychometric properties of the SDQ are strong, and recommended its use as a screening instrument (Stone, Otten, Engels, Vermulst, & Janssens, 2010). Importantly, the use of broader internalising and externalising scales from the SDQ is found to be acceptable in low-risk samples (Goodman, Lamping, & Ploubidis, 2010), has a good fit with the included subscales (Van Roy, Veenstra, & Clench-Aas, 2008), and the scales are relatively “uncontaminated” by one another (Goodman & Scott, 1999). Additionally, the prosocial scale has low correlation with the other four subscales (Goodman, 2001; Van Roy et al., 2008), supporting its exclusion from our analyses.

Accordingly, an externalising problems scale was constructed by merging the subscales of conduct problems and hyperactivity/inattention problems, while an internalising problems scale was constructed by merging the subscales of emotional problems and peer/relationship problems. We were interested in externalising/internalising symptoms that were present over time and across informants, and therefore added together the responses from teachers and parents from T1 and T2, and summed the scales to single, continuous variables. Moderate correlations between parent and teacher reports were found for both the externalising problems scale (*R* = 0.51) and the internalising problems scale (*R* = 0.47), which were evaluated as acceptable. The Cronbach’s α of the SDQ scales ranged from 0.81 to 0.85 in our sample.

#### Externalising problems

We constructed a single, continuous externalising problems variable including 2263 individuals (55.5% girls) and ranging from 0 to 57 (*M* = 9.72, *SD* = 7.86). For the purpose of secondary analyses, we also constructed a subscale for conduct problems (*M* = 2.26, *SD* = 2.75) and hyperactivity/inattention (*M* = 7.47, *SD* = 5.91).

#### Internalising problems

Similarly, we constructed a single, continuous internalising problems variable including 2266 individuals (55.4% girls) and ranging from 0 to 53 (*M* = 6.02, *SD* = 6.58). For the purpose of secondary analyses, we also constructed a subscale for emotional problems (*M* = 3.23, *SD* = 3.86) and peer/relationship problems (*M* = 2.79, *SD* = 3.71).

### Outcome variables: Alcohol- and drug-related problems

Self-reported measures of alcohol and drug use at T3 were our main dependent variables.

#### Ever tried alcohol

We used a single item “Have you ever tried alcohol?” (Yes/No) to determine whether individuals had ever tried alcohol, and the majority (*n* = 1854, 78.5%) of the sample confirmed having tried alcohol.

#### Ever tried illicit drugs

Similarly, we used another single item “Have you ever tried hash, marijuana, or other narcotic substances?” (Yes/No) to determine whether individuals had ever tried illicit drugs, and 291 (12.3%) participants confirmed that they had tried illicit drugs.

#### High-level alcohol consumption

We added up items measuring self-reported amounts of beer, cider, wine, spirits, and illegally distilled spirits usually consumed during 14 days, and each type of beverage was weighted according to its alcohol percentage. To accurately calculate alcohol consumption levels, we used data from the full sample (*n* = 10,253), and 5471 (53.3%) individuals reported any usual alcohol consumption. High-level alcohol consumption was defined as above the 80th gender-specific percentile alcohol consumption among the adolescents with any usual alcohol consumption. Based on this, a dichotomous variable was created. Within the final sample (*n* = 2438), 255 individuals reported high-level alcohol consumption, which constituted 11.4% of the sample and 18.5% of those with any usual alcohol consumption.

#### Frequent drinking to intoxication

Frequency of alcohol intoxication was measured by asking: “Have you ever consumed so much alcohol that you were clearly intoxicated (drunk)?” The original item had five response categories ranging from “No, never” to “Yes, more than 10 times”. Frequent intoxication was defined as drinking so much that one had been clearly intoxicated more than 10 times (Skogen et al., 2014), and on this basis a dichotomous variable was created. Of the participants, 439 (18.0%) reported frequent intoxication.

#### A positive CRAFFT score

Alcohol- and drug-related problems were measured using the six-item, validated CRAFFT scale. This scale has been designed to identify possible alcohol- and drug-related problems among adolescents, and has been demonstrated to have acceptable sensitivity and specificity at a cutoff of ≥ 2 (Dhalla, Zumbo, & Poole, 2011), also in the target population of our study (Skogen, Bøe, Knudsen, & Hysing, 2013). The CRAFFT scale has been found to correlate with other measures of alcohol/drug use in adolescents, supporting its efficacy as a screening tool among adolescents (Oesterle, Hitschfeld, Lineberry, & Schneekloth, 2015; Skogen et al., 2013). A dichotomous variable was calculated separating those above the cutoff of ≥ 2 on CRAFFT from those below the cutoff. We identified 499 (21.2%) participants who scored above the CRAFFT cutoff, indicating potential alcohol- and drug-related problems. In our sample the Kuder–Richardson’s reliability score of the CRAFFT scale was 0.67.

#### Total alcohol and drug use indicators

Finally, we constructed an ordinal variable for total alcohol- and drug-use indicators, summing up the number of positive scores on frequent alcohol intoxication, high-level alcohol consumption, a positive CRAFFT score, and having tried illicit drugs (Heradstveit, Skogen, Hetland, & Hysing, 2017). A total of 1435 respondents (64.5%) had none, 423 (19.0%) had one, 207 (9.3%) had two, 129 (5.8%) had three, and 30 (1.4%) had four of these potential alcohol/drug-related problems. In our sample, the Cronbach’s alpha for this ordinal scale of total alcohol/drug use indicators was 0.63.

### Included covariates

Age and gender for all participants were retrieved through personal identity numbers in the Norwegian National Population Registry. In addition, we measured self-reported perceived economic well-being by an item where participants rated their family’s economic situation either as (1) “Equal to others” (66.0%), (2) “Better than others” (28.8%), or (3) “Poorer than others” (5.2%). We also collected information on maternal and paternal educational attainment at T3 by two self-report items differentiating between primary school only, high school, or higher education. Perceived family economy, and maternal and paternal educational attainment were used as measures for socioeconomic status, and were entered separately into the regression analyses.

### Statistical analysis

#### Data preparation

The distributions on the scales for externalising and internalising problems, and their subscales, were all skewed (skewness > 1, ranging from 1.55 to 2.48 as would be expected in a community sample) (Woerner, Becker, & Rothenberger, 2004). We transformed these scales using a log transform (Rønning, Handegaard, Sourander, & Mørch, 2004), which improved the normality of the data (skewness < 1, ranging from –0.32 to 0.35). All analyses are reported on log-transformed SDQ data.

#### Descriptive statistics and regression analyses

We conducted the following statistical analyses: First, the sample was described by demographics, alcohol/drug use, and externalising/internalising problems, and was compared with the non-responders of the full BCS/youth@hordaland sample. Second, we computed odds ratios for associations between externalising/internalising problems and alcohol/drug use using logistic regression models. More specifically, unadjusted regression models were utilised, followed by adjustments for SES, gender, and age, and were adjusted for SES, gender, age, and externalising/internalising problems. Finally, unadjusted and adjusted ordinal logistic regression analyses were conducted for (a) the associations between externalising/internalising problems and ordinal number of indications on alcohol/drug-related problems, and (b) the associations between subscales of externalising/internalising problems and ordinal number of indications on alcohol/drug-related problems. All analyses were performed using STATA V.14.0 (StataCorp, 2015).

## Results

The final sample consisted of *n* = 2438 participants. Table 1 outlines the demographics of the subjects in the total included sample, as well as alcohol and illicit drug use. There were some differences between the included versus the not-included individuals, related to gender, socioeconomic status, alcohol/drug use, and externalising/internalising problems. However, effect sizes were overall small to moderate (Cohens d’s ranging from 0 to 0.35). Of note, these differences were small for externalising/internalising problems (d’s ranging from 0.18 to 0.20) and non-existent or very small for alcohol/drug use (d’s ranging from 0 to 0.09).

**Table 1.** Demographics, alcohol and drug use, and mental health problems during adolescence in the full youth@hordaland sample (*n* = 10,253)^a^.

Demographics	Study sample (*n* = 2438)	Non-included sample^b^ (n = 7815)	Cohen’s d	*p*-value^c^
Girls, %	55.3	51.8	.069	.003
Age at completion, mean	17.4	17.4	.020	.399
Perceived economic well-being, %			.043	.066
*Poorer than others*	4.4	8.0		
*Equal to others*	66.6	67.5		
*Better than others*	29.0	24.5		
Mother’s education, %			–.285	< .001
*University/college*	60.0	44.6		
*High school*	32.2	44.3		
*Primary school*	7.8	11.2		
Father’s education, %			–.345	< .001
*University/college*	56.3	38.2		
*High school*	36.3	50.0		
*Primary school*	7.4	11.8		
Alcohol and illicit drug use				
*Tried alcohol, %*	78.5	76.9	–.038	.104
*Tried illicit drugs, %*	12.3	9.6	–.089	< .001
*CRAFFT score ≥ 2, %*	21.2	21.3	.003	.908
*Frequent drinking to intoxication, %*	18.0	19.1	.029	.216
*High-level alcohol consumption, %*	11.4	12.3	.003	.298
Mental health problems^d^				
Externalising problems, mean (*SD*)	4.99 (2.98)	5.55 (3.05)	.183	< .001
Internalising problems, mean (*SD*)	4.31 (3.12)	4.98 (3.36)	.204	< .001

*Note*. *SD* = standard deviation; CRAFFT = screening tool for identification of problematic alcohol and drug use among adolescents.

^a^ All the demographic and alcohol and drug use variables listed are measured at T3. ^b^Individuals participating in the youth@hordaland study (T3) but excluded from the study sample due to not having valid Strengths and Difficulties Questionnaire (SDQ) responses at T1 and T2. ^c^ *p*-value for difference between the study sample and the sample of non-included individuals. ^d^Includes youth self-reported SDQ scores at T3.

As outlined in Table 2, externalising problems were positively associated with illicit drug use, a positive CRAFFT score, and frequent alcohol intoxication (odds ratios [*OR*s] ranging from 1.17 to 1.29, all *p* < .01) in unadjusted analyses. After adjusting for SES, gender, age, and internalising problems, externalising problems were positively associated with all measures of alcohol/drug use (*AOR*s ranging from 1.24 to 1.40, all *p* < .05). Internalising problems were negatively associated with having ever used alcohol and frequent alcohol intoxication (*OR*s ranging from 0.89 to 0.90, all *p* < .05) in unadjusted analyses. After adjusting for SES, gender, age, and externalising problems, internalising problems were negatively associated with all measures of alcohol/drug use (*AOR*s ranging from 0.83 to 0.88, all *p* < .05).

**Table 2.** Logistic regression analyses for associations between childhood externalising/internalising problems and adolescent alcohol/drug use (*n* = 2438).

	Ever used alcohol *OR* (95% CI)	Ever used drugs *OR* (95% CI)	A positive CRAFFT score *OR* (95% CI)	Frequent alcohol intoxication *OR* (95% CI)	High-level alcohol consumption *OR* (95% CI)
EXT problems					
Unadjusted	1.02 (0.93, 1.12)	1.29 (1.15, 1.45)***	1.17 (1.07, 1.28)**	1.24 (1.13, 1.37)***	1.12 (0.99, 1.25)
Adj for SES, gender, age	1.16 (1.03, 1.30)*	1.32 (1.15, 1.52)***	1.29 (1.15, 1.45)***	1.32 (1.17, 1.49)***	1.18 (1.02, 1.37)*
+ Adj for INT problems	1.24 (1.08, 1.42)***	1.36 (1.16, 1.60)***	1.32 (1.16, 1.51)***	1.40 (1.22, 1.62)***	1.27 (1.07, 1.50)*
INT problems					
Unadjusted	0.89 (0.81, 0.96)**	1.00 (0.90, 1.11)	0.96 (0.88, 1.05)	0.90 (0.82, 0.99)*	0.91 (0.82, 1.02)
Adj for SES, gender, age	0.92 (0.82, 1.02)	0.96 (0.85, 1.09)	0.97 (0.87, 1.08)	0.92 (0.82, 1.02)	0.88 (0.77, 1.01)
+ Adj for EXT problems	0.86 (0.76, 0.97)*	0.88 (0.77, 1.00)*	0.87 (0.79, 0.99)*	0.83 (0.74, 0.94)**	0.83 (0.72, 0.96)*

*Note*. *OR* = odds ratio; CI = confidence interval; EXT problems = externalising problems; INT problems = internalising problems; SES = socioeconomic status; CRAFFT = screening tool for identification of problematic alcohol and drug use among adolescents.

Bold font denotes statistically significant associations.

*p ≤ .05. **p < .01. ***p < .001.

Table 3 outlines associations between externalising/internalising problems and ordinal levels of indicators for alcohol/drug use during adolescence. Using likelihood-ratio tests of proportionality of odds across response categories, we found only non-significant differences between externalising/internalising problems and each ordinal level of indicators for alcohol/drug use in the unadjusted models (*p*-values ranging from 0.08 to 0.73), indicating that the proportional odds assumption underlying the ordinal logistic regression models was met (Liao, 1994, p. 41). We found positive associations between externalising problems and increasing levels of indicators for alcohol/drug use in both unadjusted models (*OR* = 1.21, *p* < .001) and after adjustment for SES, gender, age, and internalising problems (*AOR* = 1.38, *p* < .001). We found negative associations between internalising problems and increasing levels of indicators for alcohol/drug use after adjusting for SES, gender, age, and externalising problems (*AOR* = 0.84, *p* = .001).

**Table 3.** Ordinal logistic regression analyses between childhood externalising/internalising problems and increasing levels of alcohol and drug use indicators among adolescents (*n* = 2438).

	Increasing levels of indicators on alcohol and drug use	
	*OR*/*AOR* (95% CI)	*p*-value
Externalising problems		
* Unadjusted*	**1.21 (1.12, 1.31)**	< .001
* Adjusted for SES, gender, age*	**1.29 (1.17, 1.42)**	< .001
* Adjusted for SES, gender, age, INT problems*	**1.38 (1.23, 1.54)**	< .001
		
Internalising problems		
* Unadjusted*	0.93 (0.87, 1.00)	.057
* Adjusted for SES, gender, age*	0.93 (0.85, 1.02)	.112
* Adjusted for SES, gender, age, EXT problems*	**0.84 (0.77, 0.93)**	.001

*Note*. *OR* = odds ratio; AOR = adjusted odds ratio; CI = confidence interval; EXT problems = externalising problems; INT problems = internalising problems; SES = socioeconomic status. Bold font denotes statistically significant associations.

In secondary analyses, associations between externalising/internalising problems and ordinal levels of indicators for alcohol/drug use were analysed stratified by gender. However, no substantial gender differences were found: the same patterns of associations were evident across genders, with a similar magnitude of associations (not shown).

Table 4 sketches associations between subscales of externalising problems (conduct problems, hyperactivity) and internalising problems (emotional problems and peer problems) and ordinal levels of indicators for alcohol/drug use during adolescence. The proportional odds assumption underlying the ordinal logistic regression models was also met for these analyses (*p*-values ranging from .25 to .86). Both externalising subscales were for the most part positively associated with increasing levels of indicators on alcohol/drug use even in fully adjusted models (*AOR*s ranging from 1.19 to 1.23, all *p* < .05). Neither of the internalising subscales were significantly associated with increasing levels of alcohol/drug-related problems (*p*-values ranging from .15 to .67).

**Table 4.** Ordinal logistic regression analyses between subscales of externalising/internalising problems and increasing levels of alcohol and drug use indicators among adolescents (*n* = 2438).

	Increasing levels of indicators on alcohol and drug use	
	*OR*/*AOR* (95% CI)	*p*-value
**Subscales of externalising problems**		
Conduct problems		
*Unadjusted*	**1.16 (1.05, 1.28)**	< .001
*Adjusted for SES, gender, age*	**1.19 (1.05, 1.34)**	< .010
*Adj for SES, gender, age, hyperactivity*	1.10 (0.96, 1.25)	.168
*Adj for SES, gender, age, hyperactivity, INT problems*	**1.19 (1.03, 1.38)**	< .05
Hyperactivity/inattention		
*Unadjusted*	**1.18 (1.10, 1.28)**	< .001
*Adjusted for SES, gender, age*	**1.24 (1.13, 1.37)**	< .001
*Adj for SES, gender, age, conduct problems*	**1.18 (1.04, 1.35)**	< .010
*Adj for SES, gender, age, conduct problems, INT problems*	**1.23 (1.07, 1.41)**	< .010
**Subscales of internalising problems**		
Emotional problems		
* Unadjusted*	0.94 (0.86, 1.02)	.156
* Adjusted for SES, gender, age*	0.96 (0.86, 1.07)	.448
* Adj for SES, gender, age, peer problems*	0.97 (0.85, 1.11)	.671
* Adj for SES, gender, age, peer problems, EXT problems*	0.91 (0.79, 1.04)	.162
Peer/relationship problems		
* Unadjusted*	0.94 (0.85, 1.03)	.205
* Adjusted for SES, gender, age*	0.92 (0.82, 1.04)	.183
* Adj for SES, gender, age, emotional problems*	0.95 (0.83, 1.09)	.494
* Adj for SES, gender, age, emotional problems, EXT problems*	0.90 (0.78, 1.04)	.153

*Note*. *OR* = odds ratio; AOR = adjusted odds ratio; CI = confidence interval; EXT problems = externalising problems; INT problems = internalising problems; SES = socioeconomic status. Bold font denotes statistically significant associations.

## Discussion

Our study suggests that childhood externalising problems are positively associated, and that internalising problems are negatively associated, with adolescent alcohol/drug use and problems. These estimates were exacerbated when associations between internalising problems and alcohol/drug use were adjusted for co-occurring externalising problems, and vice versa. There was no evidence for substantial gender differences in these associations.

These findings correspond with those of a growing body of literature that is conceptualising involvement with alcohol/drug use within a broader context of antisocial or deviant behaviour (Chassin et al., 2013; Zucker, Heitzeg, & Nigg, 2011). Our results also extend the existing knowledge base of studies which have indicated that childhood internalising problems are negatively associated with alcohol/drug use in early adolescence (Colder et al., 2013; Edwards et al., 2014) to also apply to later stages of adolescence. This supports a recent contribution by Colder and colleges (2018). Also of note, both the full SDQ externalising problems scales and the subscales of conduct problems and hyperactivity/inattention were positively associated with alcohol/drug-related problems. However, the associations were somewhat stronger and more consistent in relation to the full externalising scale, possibly indicating that overall externalising behavioural tendencies are more potent predictors of subsequent alcohol/drug-related problems, compared with high symptoms on either conduct problems or hyperactivity/inattention alone.

Only the full SDQ internalising problems scale was negatively associated with alcohol/drug-related problems in our study, and only in the fully adjusted model, following the adjustment for SES, gender, age, and co-occurring externalising problems. This finding lends support to recent studies suggesting that internalising problems may protect against alcohol/drug use during adolescence (Colder et al., 2010; Hussong et al., 2011). It has been suggested that internalising problems may contribute to less alcohol/drug use due to avoidance and social withdrawal strategies, and hence potentially less exposure to situations or peer groups with high risk for alcohol/drug use (Hussong et al., 2017). Therefore, we hypothesised that peer/relationship problems were more strongly negatively associated with alcohol/drug-related problems, compared with emotional problems, but our study did not confirm this hypothesis. A possible explanation is that the influence of peer/relationship problems on alcohol/drug-related behaviours is exacerbated by emotional problems, and hence that overall internalising behavioural tendencies are more influential than peer/relationship problems alone. However, more studies are needed to clarify the complex mechanisms that link childhood internalising problems with fewer alcohol/drug-related problems during adolescence.

Although we did not find evidence that internalising problems may also be involved in “risk processes” or heighten the risk of alcohol/drug-related problems (e.g., McCarty et al., 2012; Wittchen et al., 2007), we cannot rule out this possibility. As Hussong and colleagues (2011) note in their article describing a developmental psychopathology framework for the internalising pathway to alcohol-use disorders, that a range of factors may affect the extent to which internalising problems increase the future risk of alcohol/drug use or not, such as coping expectancies and motives for alcohol/drug use, initiation of alcohol/drug use with the goal of self-medication effects, and an escalation of alcohol/drug use to the point of addiction (Hussong et al., 2011). Furthermore, it is hypothesised that the self-medication hypothesis on development of alcohol/drug-related problems becomes more relevant later in life (Virtanen et al., 2015), and therefore that internalising problems hypothetically tends to become positively associated with alcohol/drug-related problems with increasing age (Colder et al., 2013, 2018). While a study by Colder and colleagues could not confirm this hypothesis in the transition from early to late adolescence (Colder et al., 2018), a recent study by Virtanen and colleagues (2015), which followed adolescents into the adult years, clearly indicated that internalising problems predicted alcohol problems. There is a need for more studies that further investigate the potentially changing role of internalising problems on alcohol/drug-related problems from adolescence into the adult years.

### Strengths and limitations

Our study has several strengths. The sample consists of a well-defined population-based sample of children followed into adolescence, which is sufficiently large to enable a detailed investigation of longitudinal associations between childhood mental health problems and adolescent alcohol/drug use. An additional strength is the utilisation of repeated measures and multiple informants on the SDQ, providing a robust estimate for externalising/internalising symptoms.

The study has some limitations. First, although we employed a prospective design for the study with a temporal order of data collection, the findings of the study are not necessarily an expression of causality. The findings can also be explained, for example, by third factors (such as genetic predisposition, adverse life circumstances, and family characteristics), which may act as risk factors for both externalising/internalising problems and alcohol/drug use during adolescence. Additionally, we had available measures of alcohol/drug use only at T3, therefore failing to control for prior substance use. Second, complex mechanisms may be at work as mediators (e.g., timing of puberty, peer involvement, parental supervision, and parental problem drinking) between childhood externalising/internalising problems and subsequent alcohol/drug use (e.g., Dickson, Laursen, Stattin, & Kerr, 2015; Finan, Schulz, Gordon, & Ohannessian, 2015). This is beyond the scope of our study. Third, the information on alcohol/drug use was based on self-reports, while the information on childhood mental health problems was based on parent and teacher reports. The lack of clinical interviews thus adds as a further limitation to our study. However, whereas clinical diagnoses of either mental health or alcohol/drug-related problems are typically categorised as dichotomous measures, previous studies have also gained support for dimensional conceptualisations of common mental health problems (e.g., Andrews et al., 2007) and alcohol/drug-related problems (Beseler & Hasin, 2010; Krueger et al., 2004) that are not necessarily above a formal, clinical cutoff. Our study thus highlights how parent/teacher-reported externalising/internalising symptoms across a spectrum of severity are associated with self-reported alcohol/drug use. Future studies that also apply diagnostic levels of respectively externalising/internalising problems and alcohol/drug-related problems should therefore be encouraged. Fourth, although it might be preferable to have more detailed information on illicit drug use, only lifetime use was available in the youth@hordaland-survey. Illicit drug use is also particularly infrequent among Norwegian adolescents compared with other European countries (Kraus et al., 2016), and the inclusion of frequent illicit drug use could therefore be too strict a measure in the context of this study. Fifth, depression and conduct problems have previously been found to be the mental health symptoms most strongly associated with alcohol/drug use during adolescence (e.g., Armstrong & Costello, 2002; Hussong et al., 2017). In addition, associations with alcohol/drug-related problems tend to vary across subtypes of anxiety problems (Fröjd, Ranta, Kaltiala-Heino, & Marttunen, 2011; Ohannessian, 2014). However, the internalising measures of the SDQ, including its subscales, are generalised measures of internalising problems, which therefore do not separate between, for example, symptoms of anxiety and depression, and between subtypes of anxiety. Therefore, our findings may not be generalisable to all manifestations of internalising symptomatology. Sixth, only parent and teacher reports on SDQ were available at T1. Although teacher and parent versions of the SDQ have gained support in a review of psychometric studies (Stone et al., 2010), it should be noted that internalising problems are likely to be reported more accurately by children themselves (Ederer, 2004). The inclusion of self-reported externalising/internalising symptoms would add more strength to the measures of childhood internalising problems. However, there is no self-report data available on SDQ symptoms at T1 due to the young age of the children. Our externalising/internalising variables are constructed based on two time points, and thus including self-reports at one time only would not work. In addition, the use of parent/teacher reports on T1/T2 and self-reports on T3 may contribute to avoiding mono-informant bias. Finally, selective drop out is a well-known problem in longitudinal research (Wolke et al., 2009), and may not be fully ruled out in our study. However, the discrepancy between T1 (*n* = 7007) and the study sample (*n* = 2438) is not merely a result of dropout at each consecutive wave, but is also an administrative issue of the longitudinal survey design. More specifically, the BCS (comprising T1 and T2) had a different target population than the youth@hordaland (comprising T3). Therefore, it is not possible to calculate a strict attrition rate for the present study. We did, however, find some differences between the study sample and the full youth@hordaland sample, suggesting that the included individuals had somewhat higher SES than the non-included individuals. While this was the case, effect sizes for differences between the study sample and the full youth@hordaland sample were for the most part non-significant on alcohol/drug use, and were small on externalising/internalising problems. Hence, selective dropout is not likely to be an issue that would seriously bias our findings.

## Conclusion

An important contribution from our study is that childhood externalising problems are positively associated while internalising problems are negatively associated with alcohol/drug use and problems in late adolescence. Associations with alcohol/drug-related problems were similar across both genders, and were most consistent and robust when associations between externalising problems and alcohol/drug-related problems were accounted for co-occurring internalising problems, and vice versa.
